# Bats enhance their call identities to solve the cocktail party problem

**DOI:** 10.1038/s42003-018-0045-3

**Published:** 2018-05-03

**Authors:** Kazuma Hase, Yukimi Kadoya, Yosuke Maitani, Takara Miyamoto, Kohta I Kobayasi, Shizuko Hiryu

**Affiliations:** 1 Faculty of Life and Medical Sciences, Doshisha University, 1-3 Tatara miyakodani, Kyotanabe, Kyoto 610-0321 Japan; 2 Research Fellow of Japan Society for the Promotion of Science, 5-3-1 Kojimachi, Chiyoda-ku, Tokyo 102-0083 Japan; 3 JST, PRESTO, 4-1-8 Honcho, Kawaguchi, Saitama 332-0012 Japan

## Abstract

Echolocating bats need to solve the problem of signal jamming by conspecifics when they are in a group. However, while several mechanisms have been suggested, it remains unclear how bats avoid confusion between their own echoes and interfering sounds in a complex acoustic environment. Here, we fixed on-board microphones onto individual frequency-modulating bats flying in groups. We found that group members broaden the inter-individual differences in the terminal frequencies of pulses, thereby decreasing the similarity of pulses among individuals. To understand what features most affect similarity between pulses, we calculated the similarity of signals mimicking pulses. We found that the similarity between those artificial signals was decreased most by manipulation of terminal frequency. These results demonstrate that the signal jamming problem is solved by this simple strategy, which may be universally used by animals that use active sensing, such as echolocating bats and electric fish, thereby transcending species and sensory modalities.

## Abstract (web summary)

Kazuma Hase et al. use microphones mounted directly on bats flying in groups to understand how they avoid confusing the echolocation signals of multiple individuals. They find that bats manipulate the terminal frequencies of their signal pulses to decrease the similarity in pulses between individuals.

## Introduction

Animals use acoustic signals to communicate with conspecifics^1^, attract females^2, 3^, or detect food by hearing prey-generated sounds^4, 5^. When acoustic communication occurs among a large group of individuals, multiple sound sources produced by conspecifics create a complex auditory scene, presenting what is known as the cocktail party problem^6–9^. A similar situation can occur when multiple individuals of a species using active sensing emit signals to scan the surrounding environment. For example, in the presence of conspecifics, weakly electric fish create differences in the frequencies of their self-generated electric fields to solve the signal jamming problem; this is called the jamming avoidance response (JAR)^10^. However, although several mechanisms have been suggested, it is still incompletely understood how the cocktail party problem is solved by other animal species that use active sensing, such as echolocating bats. Bats use the echoes of self-generated acoustic signals to hunt, navigate, and orient themselves in total darkness. Bats also hunt or navigate with a number of conspecifics^11–13^. Because echolocating bats actively emit signals to scan their environments, groups of bats flying together experience acoustical interference caused by echoes from irrelevant directions and signals belonging to conspecifics^14, 15^. Under such circumstances, they need to extract biologically relevant sounds from noise and process them to avoid obstacles or to capture food. Understanding the acoustic behavior of group-flying bats would help to reveal how animals acquire acoustic information of interest in a complex auditory environment.

Within the same bat species, the acoustic characteristics (e.g., intensity, bandwidth, and duration) and emission timing of echolocation pulses are generally similar. For instance, identification at the species level is based on the acoustic characteristics of echolocation pulses^16^. Therefore, when multiple other individuals are flying in the vicinity, echolocating bats must extract their own echoes from others that have similar characteristics in both the time and frequency domains. However, echolocation pulses emitted by bats exhibit a certain degree of difference among individuals^17, 18^. Bats can use these differences to discriminate the echolocation pulses of individual bats^19^. It has been speculated that echolocating bats broaden inter-individual differences, e.g., the terminal frequencies, duration, and/or sweep rate of emitted sounds, to avoid confusing their own sounds with those of conspecifics^20^. However, while most previous studies have focused on changes in the acoustic features of pulses under acoustic interference, there have been no studies in which the inter-individual differences between pairs of individuals were directly measured, i.e., by utilizing on-board microphones, which can separately measure the pulses of bats flying in groups.

JARs have been reported in many species of bats. For instance, previous studies demonstrated that *Tadarida* bats flying in the field shifted their terminal frequencies in response to other bats or to echolocation pulses of the same species presented through a speaker^21^. These findings indicate the possibility that echolocating bats change the acoustic characteristics of their emitted pulses in the presence of pulses from other bats. To the best of our knowledge, however, no previous report has directly measured pulses emitted by each bat flying in a group of more than two individuals. The conventional recording methodology with fixed microphones can be used to identify individuals, especially over short distances, but one must take into account that the recorded sounds will be distorted to some extent (e.g., by the Doppler effect and atmospheric attenuation). Recently, we have used miniature on-board microphones, which measure the sounds of bats without distortion, to investigate echolocation pulses emitted by flying bats under acoustic jamming conditions; we have shown that frequency-modulating bats shift their terminal frequencies during flight depending on the frequency of presented pulse mimics^22, 23^. This technique can directly capture how each bat flying in a group changes its pulse characteristics to avoid jamming, and the present study is the first that we are aware of to demonstrate experimentally the relationship between the terminal frequencies of pairs of individuals during group flight. We found that the bats broadened inter-individual differences in terminal frequency during group flight and that the similarity of pulses between individuals in a group decreased in group flight. Our computation also revealed that the similarity between bat-like frequency-modulated signals decreased the most with manipulation of terminal frequency. The results suggested that echolocating bats flying in groups broaden the inter-individual differences in terminal frequency in order to decrease the similarity of pulses between individuals. This frequency-shifting jamming avoidance response may be universally used by animals using active sensing, such as echolocating bats and electric fish, transcending species and sensory modality borders.

## Results

### Groups of bats broaden differences in terminal frequency

**Fig. 1:**
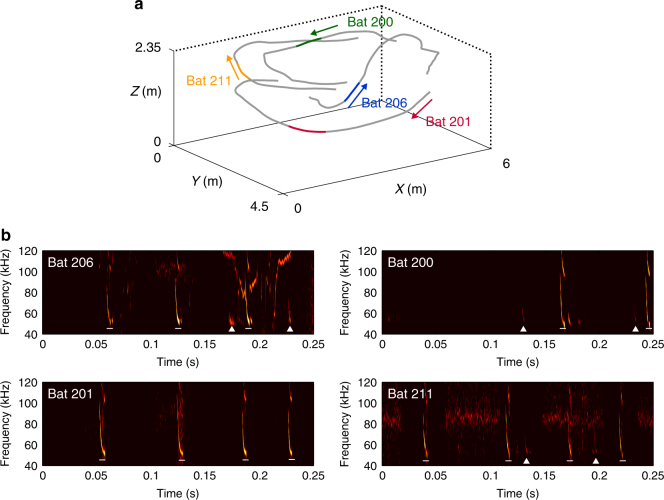
Echolocation behavior of four bats flying together. **a** Flight trajectories of four simultaneously flying bats over a 5-s period. The colors indicate different bats (ID: 206, 201, 200, and 211). The arrows indicate the flight directions. **b** Spectrograms of pulses emitted by four bats during the 250-ms intervals indicated in a as colored lines on the trajectories. White bars indicate self-generated pulses, and white triangles indicate pulses from other bats

To understand how group-flying bats adapt their echolocation behavior in response to acoustic jamming by the pulses of conspecifics, we measured the echolocation behavior of *Miniopterus fuliginosus* flying individually and in groups of four bats. We created six groups of four bats by randomly assigning 19 bats to groups, with some overlaps (Supplementary Table 1). The bats were subjected to three experimental conditions: single flight 1, group flight, and single flight 2. We used miniature on-board microphones (Telemikes) to capture the echolocation pulses emitted by each bat. Video recordings were made in order to reconstruct the three-dimensional coordinates of each bat during flight. We successfully recorded the echolocation pulses and flight trajectories of individual bats when all four bats in a group were flying together in the same flight space (Fig. 1). The bats continued flying (without landing) because they were not trained to perform any particular behavioral task. During group flights, no bat ever collided with another individual (Fig. 1a). Figure 1b shows spectrograms of the echolocation pulses emitted by each individual bat during the 250-ms time intervals indicated by the colored lines on the trajectories shown in Fig. 1a. The pulses of other bats flying nearby were occasionally recorded by the Telemikes (white triangles in Fig. 1b). We examined how the bats changed their pulse characteristics in group flights in comparison with single flights.

**Fig. 2:**
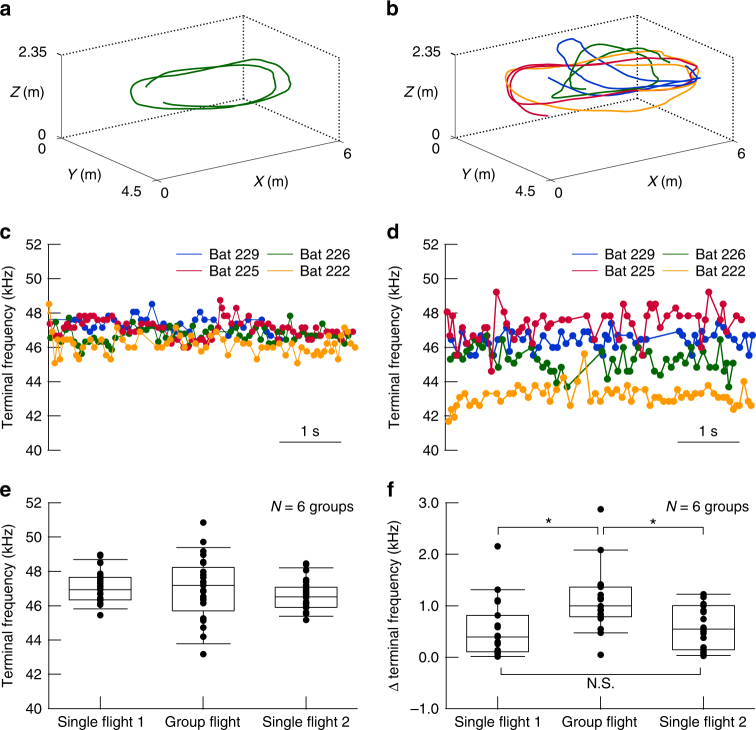
Echolocating bats use different terminal frequency channels during group flight. **a**, **b** Flight trajectories of a bat during single flight 1 and four bats during group flight for group 1. **c**, **d** Changes in the terminal frequencies of pulses emitted by four bats during single flight 1 and group flight. **e**, **f** Changes in acoustic characteristics of pulses emitted by bats during single flight 1, single flight 2, and group flight. The horizontal lines inside the boxes show the medians. The upper and lower bounds of the boxes show first and third quartile, respectively. The horizontal bars above and below the boxes show the 10th and 90th percentiles, respectively. **e** Mean terminal frequencies of each bat of all six groups in single flights 1, 2, and group flight. The data were collected from six groups (19 bats), and we obtained 24 data points per flight condition. **f** Δterminal frequencies of all six groups in single flight 1, single flight 2, and group flight. We collected three Δterminal frequencies per group, which yielded 18 data points per flight condition. The mean terminal frequency did not differ among flight conditions (one-way ANOVA, *P* *=* 0.365). Δterminal frequencies were significantly increased in group flight compared with single flights 1 and 2 (Tukey’s HSD test, *P* < 0.05)

Figure 2a–d show data from one specific group, presenting the flight trajectories and terminal frequencies of the pulses emitted by four bats during single flight 1 and group flight in a span of 5 s. Figure 2c, d show the terminal frequencies of echolocation pulses emitted by the bats during single and group flight as indicated by the trajectories in Fig. 2a, b, respectively. Each bat flying in a group seemed to use a different terminal frequency (bat 222: 43.2 ± 0.6 kHz, bat 225: 45.3 ± 0.7 kHz, bat 226: 46.4 ± 0.5 kHz, and bat 229: 47.4 ± 0.9 kHz, Fig. 2c) whereas their terminal frequencies were similar during single flight (bat 222: 46.1 ± 0.6 kHz, bat 225: 46.7 ± 0.4 kHz, bat 226: 47.3 ± 0.5 kHz, and bat 229: 47.4 ± 0.7 kHz, Fig. 2d). Figure 2e shows the mean terminal frequencies of pulses emitted by bats in all six groups (19 bats in total) during single flight 1, group flight, and single flight 2. The bats shifted their terminal frequencies in both directions, upward and downward, in group flight compared with single flights 1 and 2. However, the mean terminal frequencies were not significantly different among flight conditions (one-way ANOVA, *P* = 0.365; the mean terminal frequencies were 47.1 ± 0.9, 47.1 ± 1.8, and 46.6 ± 0.9 kHz in single flight 1, group flight, and single flight 2, respectively). Next, we tested whether bidirectional changes in terminal frequency were caused by broadening of individual differences in terminal frequency. We defined Δterminal frequency as the difference in the mean terminal frequencies between the two bats that were closest in terms of their mean terminal frequencies. Figure 2f shows changes in the Δterminal frequencies among the three flight conditions for all groups. Bats flying in groups significantly increased their Δterminal frequencies from 0.6 ± 0.6 kHz in single flight 1 and 0.6 ± 0.4 kHz in single flight 2 to 1.1 ± 0.6 kHz in group flight (Tukey’s HSD test, *P* *<* 0.05).

**Fig. 3:**
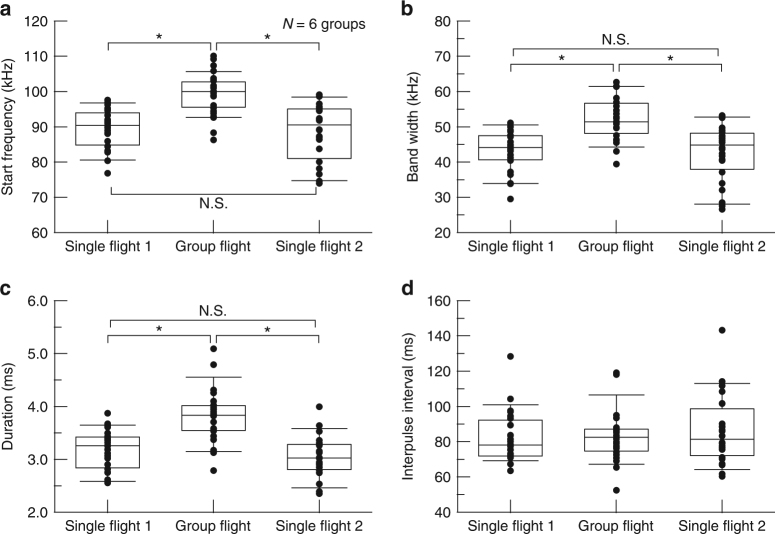
Means of the acoustic characteristics emitted by each bat in single flights 1 and 2 and group flight for all groups. **a** Changes in Fs. **b** Changes in duration. **c** Changes in bandwidth. **d** Changes in interpulse interval. We obtained 24 data points per flight condition. Horizontal lines inside boxes show medians. The upper and lower bounds of the boxes show first and third quartile, respectively. The horizontal bars above and below the boxes show the 10th and 90th percentiles, respectively. Fs, bandwidth, and duration were significantly increased from single flights 1 and 2 to group flight (Tukey’s HSD test, *P* *<* 0.05). On the other hand, IPIs did not significantly differ among flight conditions (one-way ANOVA, *P* *=* 0.695)

On the other hand, acoustic characteristics other than terminal frequencies tended to simply increase during group flight compared with single flights 1 and 2 (Fig. 3). Start frequency (Fs) increased from 89.7 ± 5.4 kHz during single flight 1 and 89.3 ± 7.8 kHz during single flight 2 to 99.3 ± 6.0 kHz during group flight (Tukey’s HSD test, *P* *<* 0.05; Fig. 3a). Bandwidth also increased from 43.3 ± 5.7 kHz during single flight 1 and 42.6 ± 7.9 kHz during single flight 2 to 52.1 ± 5.8 kHz during group flight (Tukey’s HSD test, *P* *<* 0.05; Fig. 3b). The increase in Fs was much larger than any shifts in terminal frequencies, indicating that bandwidth was increased even when terminal frequencies was shifted upwards. Pulse duration increased from 3.2 ± 0.4 ms during single flight 1 and 3.0 ± 0.4 ms during single flight 2 to 3.8 ± 0.5 ms during group flight (Tukey’s HSD test, *P* *<* 0.05; Fig. 3c). However, the interpulse interval did not significantly change. The mean IPIs were 82.4 ± 14.5 ms during single flight 1, 86.1 ± 19.4 ms during single flight 2, and 82.9 ± 14.4 ms during group flight (one-way ANOVA, *P* *=* 0.695; Fig. 3d). No coordination of emission timing during group flight was observed (Supplementary Fig. 1).

### Similarity of bat-like signals with acoustic manipulations

**Fig. 4:**
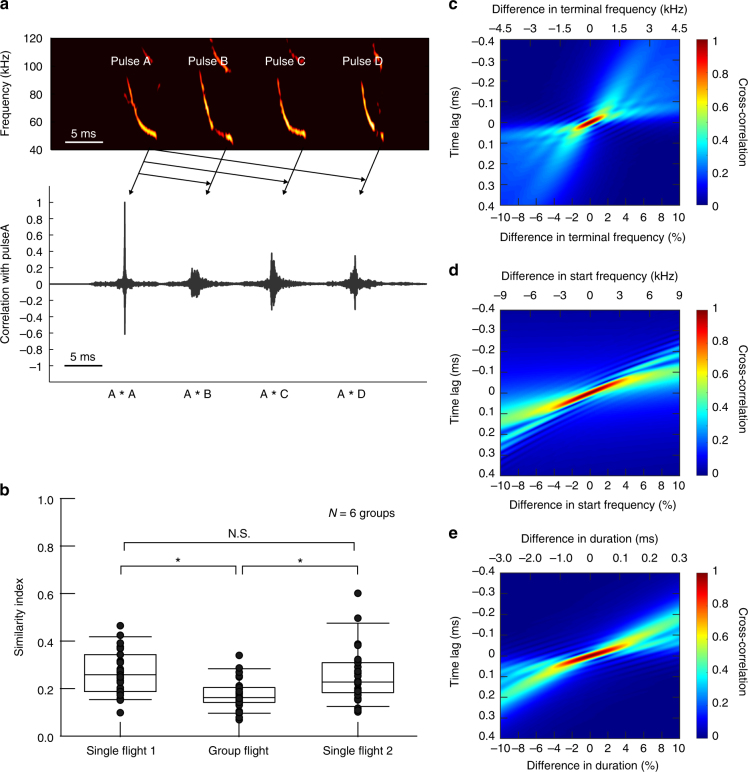
Similarities between pulses among bats of the same group and the effects of changes in acoustic characteristics on the similarities between frequency-modulated signals. **a** Spectrograms of the pulses of four bats flying together (top); correlation values of pulses A, B, C, and D with pulse A (bottom). **b** Peak cross-correlation values normalized to the autocorrelation values. We analyzed the similarity indices of all pairs of calls between four individuals in each group. This analysis yielded 36 data points per flight condition (see Methods). The upper and lower bounds of the boxes show first and third quartile, respectively. The horizontal bars above and below the boxes show the 10th and 90th percentiles, respectively. The peaks (similarity indices) significantly decreased in group flight compared with single flights 1 and 2 (Tukey’s post-hoc test, *P* < 0.05). **c**–**e** Changes in the dissimilarity function when the cross-correlation was calculated between the original frequency-modulated signal (90–45 kHz downward with a duration of 3 ms) and the manipulated signal in terms of **c** Fs, **b** terminal frequency, and **e** duration from −10 to 10%

We explored how changes in acoustic characteristics affected the similarities among bat echolocation pulses. First, to confirm that the similarity of echolocation pulses was lower in group flight than in single flight, we calculated the cross-correlations of pulses between individuals in a group when they flew singly and in the group. Cross-correlation was applied to the time-series amplitude waveforms of echolocation pulses after their amplitudes were normalized. This procedure was carried out on all the pulses that were used for sound analysis. Fig. 4a shows representative echolocation pulses emitted by four bats (pulses A, B, C, and D, respectively) during group flight, as well as the correlation values between pulse A and pulses A, B, C, and D normalized to the peak autocorrelation value of pulse A. The peak cross-correlation values with pulse A were 0.19 (vs. pulse B), 0.38 (vs. pulse C), and 0.34 (vs. pulse D) (Fig. 4a, bottom). We defined the similarity index between two bats as the peak value of the cross-correlation of their pulses normalized to the autocorrelations of pulses of either bat (see Methods). Fig. 4b shows that the similarity indices between individuals were significantly lower in group flight (0.21 ± 0.07) than in single flight 1 (0.32 ± 0.12) or single flight 2 (0.29 ± 0.12) (Tukey’s HSD test, *P* *<* 0.05).

Next, we examined which acoustic characteristics most affected similarity. We prepared a frequency-modulated signal that mimicked the echolocation pulses of *M. fuliginosus*, with an Fs of 90 kHz, a terminal frequency of 45 kHz, a bandwidth of 45 kHz, and a duration of 3 ms (Supplementary Fig. 2). Then, we calculated dissimilarity functions (i.e., cross-correlations between the signal and an acoustically modified version of the signal [in terms of Fs, terminal frequency, and duration, which we gradually changed from −10 to 10%]; see Methods). The cross-correlation values fell most when the terminal frequency of the signal was manipulated, although changes in other characteristics also resulted in decreases in cross-correlation values. Figure 4c shows that the half-width at half-maximum of the dissimilarity function was obtained when the terminal frequencies was changed by only 2%, corresponding to approximately 1 kHz (the mean terminal frequencies was approximately 48 kHz). The half-width at half-maximum was obtained when Fs was changed by 9% (corresponding to approximately 8 kHz) and the duration was changed by 7% (corresponding to approximately 0.2 ms) (Fig. 4d, e).

## Discussion

In this study, we recorded separate echolocation sounds from bats flying together in groups of four; we found that individuals shift their terminal frequencies away from those of conspecifics. The bats changed the acoustic features of their pulses when flying with multiple conspecifics. Specifically, bidirectional changes in terminal frequencies significantly broadened the differences in terminal frequency among group members (Fig. 2e). Although the direction of change in terminal frequency was almost constant within individuals, there were some exceptions (Supplementary Fig. 3). There was a tendency for individuals with lower terminal frequency to shift much lower in group flight and vice versa (Supplementary Fig. 4). Similarities in echolocation pulses among individuals were significantly decreased during group flight in comparison with single flights 1 and 2. In addition, shifts in terminal frequency were much more helpful than shifts in other acoustic features for differentiating the signal from other signals with similar characteristics, as revealed by computation of cross-correlations between a frequency-modulated signal mimicking bat pulses (original) and a copy in which an acoustic feature was changed (manipulated) (Fig. 4c). These results show that echolocating bats enhance the individual features of emitted pulses (identities) to solve the problem of signal jamming, as do other animals (weakly electric fish) that use active sensing in a different sensory modality.

*Eptesicus fuscus* bats shift their terminal frequencies upward or downward away from the frequencies of constant-frequency jamming sounds when the jamming frequencies are close to the terminal frequencies^24^. Similarly, flying frequency-modulating bats shifted their terminal frequencies when flying in pairs or in the presence of frequency-modulated jamming sounds^21–23, 25, 26^. In the present study, we observed that differences in the terminal frequencies of pulses emitted by bats during group flight were significantly greater than in single flight, and the similarities among pulses also significantly decreased in group flight compared to single flight. Taken together, the findings suggest that echolocating bats maintain or expand differences in frequency because it is important for bats to avoid spectral jamming by other conspecifics.

On the other hand, some recent studies have cast doubt on the notion that the JAR involves shifting of frequencies^18, 27, 28^. These studies suggest that the spectral shifts are caused by the physical presence of nearby individuals, rather than acoustic jamming from conspecifics. The JAR is suggested not to be attributable to shifting of frequencies, as most previous studies focused only on changes in the frequencies of emitted pulses and not on increased inter-individual differences in frequency (corresponding to the Δterminal frequencies of the present study). In the present study, there was no significant correlation between terminal frequency and the duration of emitted pulses, indicating that the observed changes in terminal frequency during group flight were not due to the changes in duration caused by the changes in distance from other individual bats flying in the group (Supplementary Fig. 5). In addition, the present study was the first to our knowledge to track and identify pulses emitted by each individual in groups of four bats without distortion. As a result, we observed bidirectional changes in terminal frequencies and decreases in the similarity of pulses among individuals during group flight; we tested the same bats in group flight and single flights scheduled before and after the group flights (Fig. 2e, f). We found that the Δterminal frequencies were smaller (approximately 1 kHz) than the inter-individual differences or flight-induced Doppler shifts (approximately 0.5–1.5 kHz in this study); thus, they may have been overlooked in previous studies because of the methodologies used.

The echolocation pulses emitted by some species of frequency-modulating bats (including *M. fuliginosus*, *Pipistrellus abramus*, and *E. fuscus*) are composed of a frequency-modulated portion that is specialized for measuring distance and a quasi-constant frequency (QCF) portion that is helpful for target detection. The bats may be able to use both aspects of compound frequency-modulated-QCF pulses to measure target distances and detect relatively distant targets. We found that even a slight difference in the terminal frequency of bat-like signals reduced the similarity between sounds. Therefore, the frequency of the QCF portion may serve as a “tag” that indicates the identity of an individual pulse and may be used by bats to discriminate their own echoes from those of others.

We also observed increases in sound intensity and peak frequency during group flight (see Supplementary Fig. 6). It has been reported that bats confronted with noises increase the sound intensity and/or frequency of their emitted pulses^18, 29^. The involuntary regulation of the intensity, pitch, and/or syllable duration of vocalizations in animals, including humans, in the presence of noise is called the Lombard effect^30–34^. The increase in the intensity of emitted pulses and the lengthening of pulse duration, which increase the concentration of energy in the low frequency range in the outgoing signals, may result in an improved signal-to-noise ratio. Moreover, longer sounds are more detectable than shorter sounds because the auditory system can integrate sound over time^35^. Our results suggested that echolocating bats emit louder and longer pulses in noisy situations to improve the signal-to-noise ratio of their returning echoes, as suggested by previous studies^18, 29^.

How do our current results apply to group flight of bats in the real world? Although shifts in frequency are helpful to avoid acoustic jamming, the shifts cannot fully explain the ability of bats to fly with enormous numbers of conspecifics in nature. Echolocating bats can use other potential solutions to avoid acoustic interference from other bats. Vocal timing is one of the most effective means to avoid confusion, especially in low-duty-cycle bats. Stationary *T. brasiliensis* reduce the number of pulses in the presence of interfering sounds and conspecifics^36, 37^. Similarly, flying *P. abramus*^22^, *T. brasiliensis*^38^ and *E. fuscus*^39^ bats regulate vocal timing in response to jamming sounds or the sounds of conspecifics in the group. Moreover, the directionality and directivity of the ears and pulses serve as spatial filters, allowing bats to focus on a point in three-dimensional space and ignore sounds that come from off-axis angles. Echolocating bats may use these mechanisms effectively to avoid jamming by the pulses emitted from other bats, allowing effective collective behavior when groups contain large numbers of bats. Our experimental design focused on addressing the mechanisms of jamming avoidance for small groups of bats flying in echoic closed spaces. It seems difficult for each bat to find an “open slot” through terminal frequency alone when a swarm of dozens of flying bats results in a chaotic acoustic environment.

We are the first, to our knowledge, to show directly that groups of bats mutually separate their frequencies to reduce the similarities between pulses of different individuals. On the basis of previous work on the JAR of electric fish, which increase frequency differences when in self-generated electric fields^10^, we suggest that animals using active sensing employ universal rules that transcend species and sensory modality boundaries. Furthermore, our calculations show that bat-like combination signals (with frequency-modulation and QCF portions) can be differentiated by slight shifts in frequency. Although it is currently difficult to correlate the results of our computations with auditory perception in bats, this simple strategy could also be used as a method of signal separation in various engineering fields, including radar or sonar research.

## Methods

### Subjects

We used 19 M fuliginosus bats (body mass, 12.6–18.1 g; 10 males and 9 females) in this study. We collected the bats from large colonies roosting in natural caves in Hyogo and Fukui prefectures, Japan. We were licensed to collect the bats, and we complied with all Japanese laws (permits from Hyogo prefecture in 2015 and from Fukui prefecture in 2016 and 2017). The animals were housed in a temperature-controlled and humidity-controlled colony room [4 (L) × 3 (W) × 2 m (H)] at Doshisha University in Kyoto, Japan. They were allowed to fly freely and had ad libitum access to food (mealworms) and vitamin-enriched water. The day-night cycle of the room was set to 12 h:12 h dark: light.

All experiments complied with the Principles of Animal Care (publication no. 86-23 [revised 1985)] of the National Institutes of Health) and all Japanese laws. All experiments were approved by the Animal Experiment Committee of Doshisha University.

### Experimental procedure

All flight experiments were conducted in an experimental chamber [9 (L) × 4.5 (W) × 2.4 m (H)] at Doshisha University in Kyoto, Japan. The chamber was constructed of steel plates to minimize interference from external electromagnetic noise and commercial frequency-modulation radio stations. There was no acoustic foam because we wanted to make the chamber more echoic and make the acoustic situation more extreme in order to elicit clear jamming avoidance behavior. During all experiments, long-wavelength lighting with filters (removing wavelengths below 650 nm) was used to avoid visual effects on the bats. Nineteen bats were randomly assigned to six groups of four bats, with some overlap (Supplementary Table 1). The bats were allowed to fly individually and in groups in a flight space [6 (L) × 4.5 (W) × 2.4 m (H)] surrounded by walls and a net suspended from the ceiling within the experimental chamber. There were no obvious landing sites in the flight space.

The bats were tested under three experimental conditions: single flight 1, group flight, and single flight 2. For each group, all flights were conducted within one day. The detailed procedure was as follows. First, each bat in a group was released by an experimenter and flew individually in the experimental chamber for approximately 30 s (single flight 1). After those single flights were recorded, each bat was kept in an individual cage. Then, two experimenters released four bats simultaneously so that they flew together in the chamber for approximately 60 s (group flight). Finally, once more, an experimenter allowed each bat in the group to fly alone in the chamber for approximately 30 s (single flight 2).

### Telemike recordings

Echolocation pulses emitted by each flying bat were recorded by a custom-made miniature on-board microphone (Telemike) mounted on the back of the bat. The details of the Telemike recording procedure have been described previously^40^. To record separately the echolocation pulses emitted by each bat flying in a group, we attached a Telemike to the back of each individual bat. The Telemike transmitted frequency-modulation radio signals using a carrier frequency between 76 and 104 MHz. We assigned a different carrier frequency to each Telemike within a group so that the transmitted signals would not interfere with each other. After the transmitted signals had been received by a frequency-modulation radio antenna (Terk Technologies Corporation, FM+, Commack, New York, USA) suspended from the ceiling of the chamber, they were demodulated using a custom-made frequency-modulation receiver (ArumoTech Corporation, Kyoto, Japan) featuring five independent channels with bandpass filters of 10–200 kHz. The signals were then digitized using a high-speed data-acquisition card (National Instruments, Model NI PXI-6358, Tokyo, Japan, 16 bit, *f*_s_ = 500 kHz). The total frequency response of the Telemike system was flat (within ±3 dB) between 20 and 100 kHz.

### Video recordings

Video recordings were made by two digital video cameras (IDT Japan, Inc., MotionXtra NX8-S1, Tokyo, Japan) running at 30 frames per second. The cameras were located outside the flight space (at two of the top corners of the chamber). The captured video images were stored on a personal computer. The two video cameras recorded a three-dimensional cube of known coordinates positioned in the center of the flight space before the flight experiments commenced. Three-dimensional reconstruction of each bat’s flight path was performed with motion capture software (Ditect Corporation, DippMotion PRO version 2.21a, Tokyo, Japan) using direct linear transformation with reference to the coordinates of the reference frame.

### Sound analysis

The number of pulses we analyzed for each bat ranged from 173 to 467. The pulse counts were different because we analyzed only those pulses that occurred when the four bats were actually flying together and the telemetry recordings had a good signal-to-noise ratio. We also excluded pulses before and after the buzz because those pulses sometimes had unusual duration and terminal frequency (they were much shorter and had lower terminal frequency). As a result, we analyzed 4.2–9.8 s (mean duration of 6.5 s), depending on the duration of time the bats spent flying in groups of four. The acoustic characteristics of echolocation pulses were manually analyzed on spectrograms from Telemike recordings using custom-written MATLAB scripts running on a personal computer. Each Telemike was intended to record the echolocation pulses and echoes of one bat. However, during group flight, the microphones sometimes recorded not only the pulses of the intended bat but also those of other bats. To extract and analyze the echolocation pulses of individual bats correctly in such cases, we visually discriminated pulses on the basis of amplitude and timing across oscillograms and spectrograms of the four recorded channels.

We defined the Fs and terminal frequency of each sound as the highest and lowest frequencies, respectively, of each pulse in the spectrogram that lay −25 dB from the maximum energy portion of the spectrogram. The duration was also determined from the spectrogram, at −25 dB relative to the maximum energy portion. The bandwidth was calculated by subtracting the terminal frequency from the Fs. We defined a neighboring bat as the bat with the nearest mean terminal frequency to a bat in the same group. Δterminal frequency was defined as the difference in mean terminal frequency between a bat and the neighboring bat.

All statistical analyses were performed using SPSS version 24 (IBM, Armonk, New York, USA). We employed one-way ANOVA to test whether the terminal frequency, Δterminal frequency, Fs, bandwidth, duration, interpulse interval, or cross-correlation peak values differed significantly among the three flight conditions (single flight 1, group flight, and single flight 2). If the main effect was significant, we then applied Tukey’s post-hoc test. *P*-values <0.05 were considered significant. The results are presented as the means ± SDs.

### Similarity index

To explore whether the similarities of pulses among individuals in a group changed significantly, we used cross-correlation as a similarity index. Cross-correlation values were calculated between the pulses of each pair of individuals in each group, creating six combinations per group (_4_C_2_ per group). Cross-correlation was applied to the time-series amplitude waveforms of echolocation pulses after their amplitudes were normalized. This procedure was carried out on all the pulses that were used for sound analysis. For example, to obtain the similarity index between bat A and bat B in single flight 1, we conducted cross-correlation for all pairs of calls between bat A and bat B during the flight (for example, bat A and bat B of group 1 produced 128 and 152 pulses, respectively). The obtained cross-correlations of any combination were normalized to the autocorrelation values of the pulses of either bat. We defined the similarity index as the mean of the peak values of the normalized cross-correlations between two individuals. This analysis yielded 36 similarity indices (_4_C_2_ combinations × six groups) per flight condition (single flight 1, group flight, and single flight 2).

### Dissimilarity function

We created frequency-modulated signals modulated from 90 to 45 kHz over a 3-ms period, mimicking the pulses of *M. fuliginosus*. We calculated cross-correlations between the original signals and the acoustically modified signals when each acoustic characteristic (Fs, terminal frequency, and duration) was gradually changed from −10 to 10%. The calculated cross-correlation values were plotted as functions of the modifications in the acoustic features. The half-width at half-maximum values of the dissimilarity functions showed how changes in an acoustical feature affected the degree of similarity between the two signals.

### Data availability

All sound data analyzed in the current study are available from Dryad at 10.5061/dryad.4f99c46^41^.

## Electronic supplementary material

Supplementary Information
