# Supplementary material for: Bats enhance their call identities to solve the cocktail party problem
Source: Commun Biol. 2018 May 3;1:39. doi: 10.1038/s42003-018-0045-3 (PMC6123623; doi:10.1038/s42003-018-0045-3)
Supplement: Supplementary file 1 — Supplementary Information [file 42003_2018_45_MOESM1_ESM.pdf]

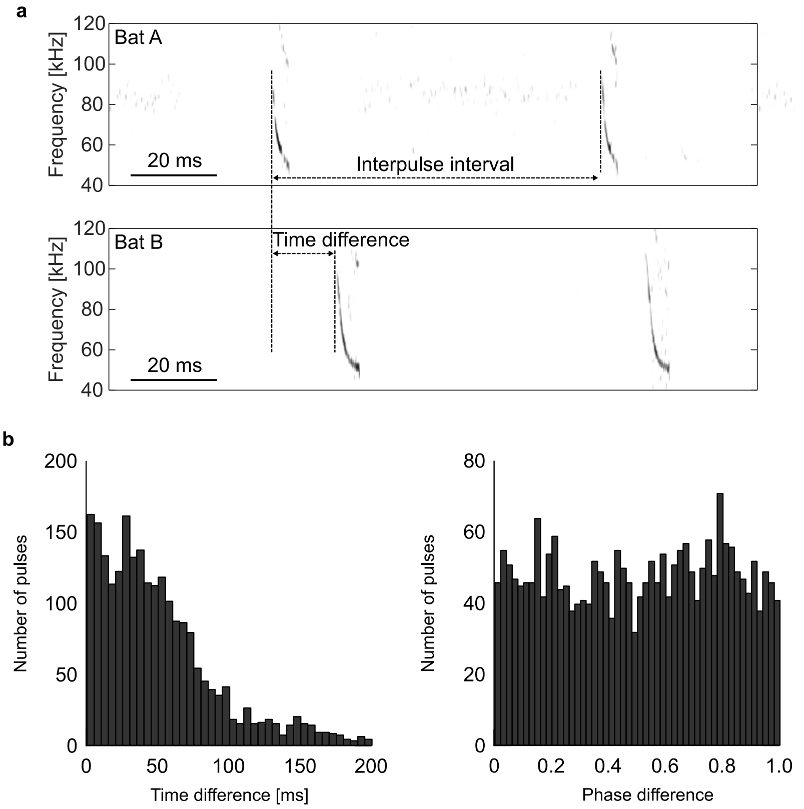

Supplementary Figure 1 | Emission timing of echolocation pulses during group flight. a, Definitions of the time difference and phase difference. The time difference is the difference between the emission time of one bat and those of other bats. The phase difference is obtained by dividing the time difference by the interpulse interval. We calculated the time difference and the phase difference of each possible pair of bats in each of the six groups. **b**, Histograms of time difference (left) and phase difference (right).

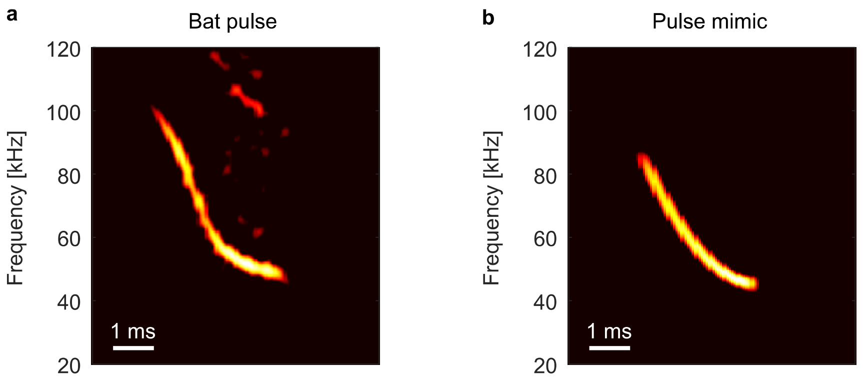

Supplementary Figure 2 | Typical echolocation pulse of *M. fuliginosus* and pulse mimic. a, Spectrogram of a typical echolocation pulse emitted by *M. fuliginosus* recorded with a Telemike. **b**, Spectrogram of a pulse mimic for calculation of the dissimilarity function. The generated pulse had an Fs of 90 kHz, a terminal frequency of 45 kHz, and a duration of 3 ms.

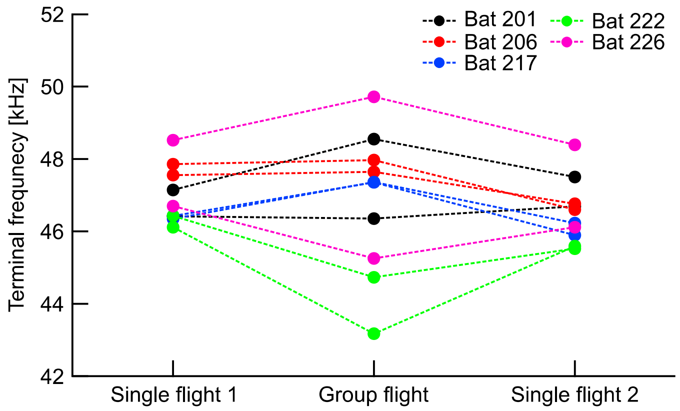

Supplementary Figure 3 | Directions of changes in terminal frequency by the same bats in different group flights. Changes in the mean terminal frequency of the bats that experienced group flight twice in single flight 1, group flight, and single flight 2. Different colors indicate different bats. Although the bats tended to shift their terminal frequency in the same directions in two different group flights, there were some exceptions (e.g., Bat 201 and Bat 226).

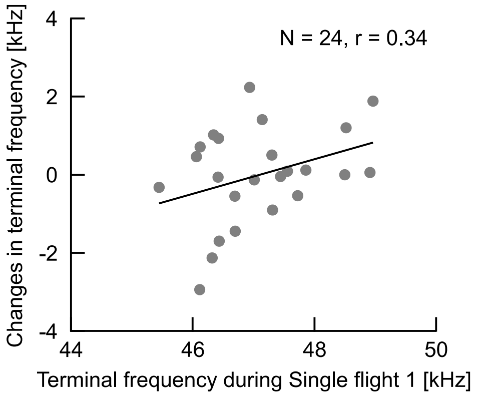

Supplementary Figure 4 | Relationship between changes in terminal frequency and terminal frequency during single flight 1. The plot shows changes in mean terminal frequency and mean terminal frequency during single flight 1. The solid line is a regression line. Individuals with lower terminal frequency shifted much lower in group flight, and vice versa. There was a weak correlation between mean terminal frequencies during single flight 1 and changes in terminal frequency from single flight 1 to group flight (r = 0.34).

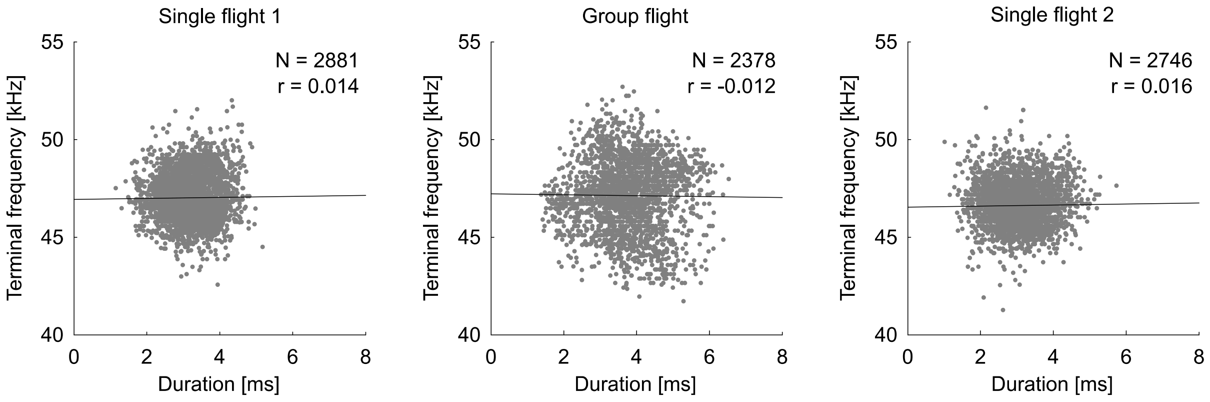

Supplementary Figure 5 | Correlation of terminal frequency with duration of emitted pulses. Black lines indicate regression lines. The data did not show a correlation between **terminal frequency** and duration in single flight 1 (left), group flight (middle), or single flight 2 (right), indicating that the observed changes in **terminal frequency** were not due to the changes in duration caused by the changes in distance from other individual bats flying in the group.

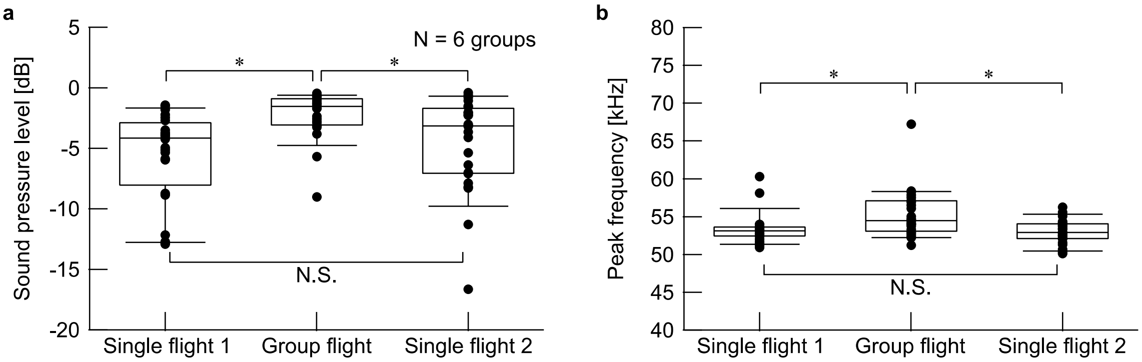

Supplementary Figure 6 | Mean acoustic characteristics of pulses emitted by each bat in each group in single flights 1 and 2 and in group flight. a, Changes in SPL. **b**, Changes in peak frequency. SPL was calculated from the peak-to peak-amplitude voltage of each pulse in the time domain. A value of 0 dB was defined as the maximum value of pulses recorded among all flight conditions. We plotted 24 data points per flight condition. The horizontal lines inside the boxes show the medians. The upper and lower margins of the boxes show the first and third quartiles, respectively. The horizontal bars above and below the boxes show the 10th and 90th percentiles, respectively. SPL and peak frequency were significantly increased from single flights 1 and 2 to group flight (Tukey's HSD test, $P < 0.05$).

Supplementary Table 1 | The IDs of the four bats in each group and their terminal frequencies (means ± SDs) during single flight 1.

Group	Bat ID and terminal frequency [kHz]			
1	DBT0200<br>45.4 ± 0.8	DBT0211<br>46.1 ± 0.9	DBT0201<br>47.1 ± 0.5	DBT0206<br>47.6 ± 1.1
2	DBT0201<br>46.4 ± 0.8	DBT0208<br>47.7 ± 1.0	DBT0206<br>47.9 ± 0.7	DBT0202<br>49.0 ± 1.1
3	DBT0222<br>46.1 ± 0.6	DBT0225<br>46.7 ± 0.4	DBT0226<br>47.3 ± 0.5	DBT0229<br>47.4 ± 0.7
4	DBT0217<br>46.3 ± 1.4	DBT0220<br>48.5 ± 0.6	DBT0226<br>48.5 ± 0.8	DBT0224<br>48.9 ± 0.7
5	DBT0223<br>46.3 ± 0.4	DBT0222<br>46.4 ± 0.5	DBT0217<br>46.4 ± 0.6	DBT0228<br>46.7 ± 0.7
6	DBT0247<br>46.1 ± 0.6	DBT0248<br>46.9 ± 0.5	DBT0240<br>47.0 ± 0.5	DBT0027<br>47.3 ± 0.5
